# Healthcare staff's views on the patients' prerequisites to be co‐creator in preventing healthcare‐associated infections

**DOI:** 10.1111/scs.12730

**Published:** 2019-06-28

**Authors:** Elisabeth Berglund Kristiansson, Ulrika Källman

**Affiliations:** ^1^ Department of Public Health Science University of Skövde Skövde Sweden; ^2^ Research Department Region Västra Götaland, South Älvsborg Hospital Borås Sweden

**Keywords:** health care‐associated infection, patient safety, patient involvement, patient participation, co‐creator, qualitative research and qualitative content analysis

## Abstract

**Background:**

Every year, patients are affected by suffering and death caused by adverse events in connection with health care and the most common adverse events are healthcare‐associated infections (HAI). The Swedish Patient Act from 2015 strengthens the patient's position in health care; however, there is lack of knowledge of how healthcare staff experience the possibilities to make the patient involved in the preventive work of HAI.

**Aim:**

To describe healthcare professionals' views on the patient's prerequisites to be co‐creator in preventing HAI in connection with hospital care.

**Method:**

This study had a qualitative descriptive design with semi‐structured individual interviews. Qualitative inductive content analysis was used to analyse the transcribed interview data. The study setting was a hospital in Sweden in 2015. Interviews were carried out among six healthcare professionals.

**Results:**

In the analysis, 5 themes and 14 categories were identified in three different domains: *Organisation, Healthcare staff and Patient.* The result indicates an obstacle in each domain for the patient to become co‐creator in preventing HAI. In *Organisation* domain, a lack of organizational structure such as elaborated working methods to involve the patient was pointed out. In the domain *Healthcare staff,* it showed that the professionals allocate the responsibility of preventing HAI to the patient but only if they had to or if they trusted the patient. In the *Patient* domain, the result states that the patient was perceived as passive; they did not take own initiatives to get involved.

**Conclusion:**

The patient has an important role in successful HAI prevention work and should be considered as an obvious co‐creator. Nevertheless, this study shows that neither organisation nor healthcare staff are sufficiently prepared for this. The organisation must make an anchored, structured and systematic work centred on the patient's needs and give more support both to healthcare professionals and patients.

## Introduction

Every year, patients in both Sweden and other countries are affected by suffering and death caused by adverse events in connection with health care. According to the Swedish Patient Safety Act, adverse events can be avoided if adequate actions have been undertaken 1.

The most common adverse event is healthcare‐associated infections (HAI) with high prevalence of wound and urinary tract infections 2, 3. The injuries cause human suffering and have a huge impact on health care in general with prolonged care times and increased workload for the healthcare staff 4.

Calculations show that between 3.2 and 4.5 million people in Europe are afflicted with HAI and about 37 000 of them die 5. In Sweden, about 65 000 adult in‐hospital patients are estimated to be afflicted with HAI; about 1500 of them die and about half of these events and deaths are considered to be avoidable 2. This means that most of these events could have been prevented. However, even though lots of interventions to prevent HAI have been undertaken in health care during the past decade, the prevalence of HAI is still high; this is, of course, unacceptable.

In a report from 2014, the Swedish Association of Local Authorities and Regions (SALAR) highlights successful factors in the prevention of HAI, all based on the organizational structure of healthcare professionals and management 6. However, they emphasise that the patient also has a significant role in the success and should therefore be given a co‐creating role in all work in the prevention of, for example, HAI.

Vaismoradi and colleagues 7 elucidate five areas that are important for enabling the patient as a co‐creator in patient safety work. These areas are, apart from the *patient*, the task needed to be undertaken, *healthcare provider*, *work environment*, and *organisation and management*. They point out that the patients' health condition, knowledge and previous experience of the healthcare system influence their ability to actively participate in preventive initiatives, as well as their beliefs and attitudes to the healthcare staff. In parallel, the attitude of the healthcare staff and their trust of the patients also affects the opportunities for patient participation and how the task is perceived. If the patient starts to mention errors in safety practices, it could be perceived as a compliant and experienced negatively. The work environment is a further prerequisite for patient participation in safety care; Vaismoradi and colleagues conclude that an open atmosphere is important, that role models are provided, and that patient participation is valued. Finally, they emphasise the importance of a management and organisation that provides the necessary resources and infrastructures for patient participation, which could increase the ability for the patient to be a co‐creator in preventing HAI.

In January 2015, the Patient Act was introduced in Sweden 8. The law supports the patient's position in health care and stresses the importance of the patient participating in the healthcare providers' patient safety work. However, there is a lack of knowledge of how healthcare staff experience the possibilities to make the patient involved and a co‐creator in the preventive work.

### Aim

To describe healthcare professionals' views on the patient's prerequisites to be co‐creator in preventing healthcare‐associated infections (HAI) in connection with hospital care.

## Method

### Design

This study had a qualitative descriptive design with semi‐structured individual interviews.

### Setting

The study was conducted in a hospital in southern Sweden during the spring of 2015. At the time of the study, the hospital had approximately 400 beds divided into 23 departments. The interviews were carried out among healthcare professionals within orthopaedic, surgery and medicine clinics.

### Data collection and participants

Healthcare professionals were eligible for participation if they worked on a daily basis in somatic patient care and were either a physician, nurse or assistant nurse. Hygiene experts were excluded. Six healthcare professionals (two physicians, two nurses and two assistant nurses) were invited. Five women and one man were included, ranging in age from 26 to 65 years. They had worked from 9 months up to 38 years.

The interviews were conducted at the participant's workplace and started with a reading of the purpose of the study. The interviews began with an open‐ended question: ‘Linked to the purpose of this study, can you please tell me about the work which is done to prevent HAI?’ followed by ‘can you tell more about patients' prerequisites to be participants in preventing HAI?’. When necessary, the informants were asked to clarify what they meant, or follow‐up questions were posed, such as: ‘can you explain more?’ or ‘what do you mean?’. The interviews, which lasted for 45–60 minutes, were tape‐recorded and transcribed verbatim 9.

### Data analysis

Using a qualitative inductive content analysis 10, all the interviews were first read several times to obtain an overall sense of the interviews. Thereafter, meaning units corresponding to the purpose of this study were identified. These meaning units were then condensed, abstracted and coded. The codes were compared for similarities and differences and grouped into different categories. The categories that related to each other were linked under one theme and sorted into three different domains. Examples of the content analysis process from this study are shown in Table 1.

**Table 1:** An example of the qualitative content analysis process in this study

Meaning unit	Condensation	Code	Category	Theme
It is mentioned … that it pays to quit smoking 6 weeks before surgery to prevent sore infection	Mention that it pays to quit smoking to prevent sore infection	Recommend quit smoking	The healthcare staff informs and explains	The healthcare staff have one‐way communication
If you stay for another day you will get pneumonia, I used to say (to motivate the patient to participate in preventing HAI). As a pure scare propaganda. That´s what people believe in	Scares the patient to get up to avoid pneumonia	Scares the patient	The healthcare staff exhort and enforce	

## Results

In the analysis, 5 themes and 14 categories were identified in three different domains: *Organisation, Healthcare staff and Patient* (Table 2). The results are presented below based on these themes and categories within each domain and illustrated with quotations from the interviews.

A sense of the whole is that the staff do not usually think of the patient as a co‐creator in preventing HAI. Instead, they see the patient more as a recipient of care and information.

**Table 2:** Overview of the results

Category	Theme	Domain
The working methods are unstructured Person‐related factors determine whether the patient is involved It is new in the HAI‐prevention work that the patient is co‐creator	The organisation lacks structure to empower the patient as co‐creator	The organisation
Varied trust in the patient The healthcare staff both give the patient responsibility and take over The healthcare staff keep a balance between profit and risk	The healthcare staff allocate responsibility	The healthcare staff
The healthcare staff inform and explains The healthcare staff exhort and enforce The healthcare staff adjust their communication to reach out	The healthcare staff communication is a prerequisite	
The patient does not take their own initiatives The patient ignores the consequences	The patient is passive	The patient
The patient´s interest and resources are important The patient's previous knowledge is at different levels Will and resistance of the patient	The patient has possibilities and limitations	

### The organisation

#### The organisation lacks structure to empower the patient as co‐creator

This theme is characterised by the fact that the informants revealed that *working methods are unstructured* and *person‐related factors determine whether the patient is involved* in preventing HAI or not. They point out that there is no given structure for when and if the patient is to be involved in preventing HAI. In addition, work situation and time pressure are factors which influence whether the staff have time for or remember to inform the patient.

The healthcare staff perceive *it is new in the HAI‐prevention work that the patient is co‐creator.*

I have never thought of that. To get the patient involved. It's new … I think it's very new to the patient (Participant 6)

Furthermore, they reflected that preventive measures to counter HAI are conducted but not always explained to the patient. The staff assess on a case‐by‐case basis which patients are to be involved but a certain scepticism was expressed about the importance of providing information in all situations.

… they are worried about every little thing and then maybe you should refrain from informing them. Because whether you are here or not, you are exposed to bacteria and infections more or less (Participant 2)

### The healthcare staff

#### Healthcare staff allocate responsibility

In this theme, the healthcare staff's *varied trust in the patient* is described and the fact that *the healthcare staff both give the patient responsibility and take over* the prevention of HAI. It is emphasised that the *healthcare staff keep a balance between profit and risk* in the allocation of this responsibility because they do not always trust the patient's understanding and they are therefore reluctant to relinquish their responsibility. At the same time, they sometimes need to trust the patient because they feel they could not always control everything.

Then you can't take over the patient altogether as much as you want (Participant 1)

Some informants expressed that the healthcare staff hand over the responsibility to the patient concerning the prevention of HAI when instructing them to shower before surgery according to given instructions or when to contact the healthcare provider if they have wounds.

… the hibiscrub showers before surgery … they do it in the evening before and in the morning before they come and that is something we make them do but… elective [surgery] patients … (Participant 1)

The patient may also take responsibility for HAI‐prevention work when the discussion is in progress before a surgical intervention and the patient must also assess the benefit of the operation compared to the risk of an infection.

… when we recommend a pacemaker to the patient … then we always discuss the risk of infection. You are discussing the benefit …. (Participant 6)

It was stated that in some care situations, it is necessary to take over and control the patient. Examples of such situations could be decisions about prophylactic treatment with antibiotics to prevent postoperative wound infection or a decision to insert a urinary tract catheter if the patient becomes worse and is urinating poorly. Healthcare staff do not consider care as the patient's responsibility and therefore do not give the patient the opportunity to express his or her own choices. The patient is seen as a recipient of care.

… in some situations, we always give prophylaxis (antibiotics). Then we say we do, it's not an issue (Participant 5)

Sometimes, the healthcare staff find that they balance between profit and risk when they, on one hand, want the patient to participate in the HAI prevention work, but on the other hand, the patient will be exposed to a risk unless the patient does not take the right action. They want to be flexible to the patient, but they point out that it can be difficult to involve the patient in the continuous work of hygiene. Sometimes, healthcare professionals can feel dejected and it can be hard to understand the patient. At the same time, it was stated that they would not give up for the patient in the HAI prevention work.

No, it's not always easy to motivate … then you have to try the next day … do not give up … someone else may try … it can go better (Participant 4)

#### The healthcare staff communication is a prerequisite

Different forms of communication given from the healthcare staff were highlighted as a prerequisite for the patient to become a co‐creator. The first entails that *the healthcare staff inform and explain* to the patient, the second entails that *the healthcare staff exhort and enforce* patient, and the last approach is about the *healthcare staff adjusting their communication to reach out to* the patient.

An important part of the work to prevent HAI is informing the patient about what they have to do and explain why. This could include both providing information before hospitalisation and during the care period at the hospital. Before hospitalisation, the information could be about the importance of showering with bactericidal soap or to quit smoking before surgery to reduce the risk of wound infection.

It says … that it pays to quit smoking 6 weeks before surgery to prevent wound infection. (Participant 5)

Information that was considered important to share with the patient during the care period could be to explain the importance of early postoperative mobilisation to prevent pneumonia, urinate at toilet to prevent urinary tract infections and eat and drink to counter the onset of wound infection.

In addition to informing and explaining, interviewers argued that they sometimes felt that there was a need to directly exhort and force the patient for their own activity, thus giving them the opportunity to be co‐creators. Someone pointed out that it was considered important to encourage the patient to be active and ask for pain relief to avoid aching after surgery and thus making it easier to get up and exercise. Furthermore, some informants expressed that they also chose to scare the patient into activity by telling them about the risks of infection if they lay in bed for too long.

If you stay one more day in bed, you will get pneumonia, I usually say (to motivate the patient to participate in preventing HAI). As a pure scare propaganda. People actually believe in that. (Participant 5)

Other methods of forcing patients to physical movement could be to discontinue the urinary tract catheter when deemed appropriate, even if the patient does not want to because he or she wants to remain in bed. Other patients were exhorted to wash their body thoroughly to avoid infections.

Some men who have chronic (urinary) catheters … it's good to tell them to wash carefully, because men are a little sloppy. … yes around, pull down your foreskin and so. (Participant 3)

During an interview, the informant raised the issue of the healthcare staff using the patient's language to communicate; this means that medical terminology is avoided when communicating with the patient. Furthermore, it was emphasised that both word choice and tone, as well as brief and directed information, were considered important for the patient to understand.

… and it is very important, and yes, from my side, it is very important that the information is short and concise so that they understand. (Participant 2)

The directed information could be in the form of written instructions for self‐care or short, targeted, written messages placed in selected places to enable participation in preventing HAI.

… patch on the mirrors … wash your hands … easy way to influence the patient … exhortation … (Participant 1)

### The patient

#### The patient is passive

The patient is passive theme includes the categories *the patient does not take his/her own initiatives* and *the patient ignores the consequences*.

The informants' experience was that the patient rarely questioned the care given and surrendered to the care and healthcare staff and assumed that they were taken care of.

I do not think they think … you get to a hospital and then you assume that such things (preventing HAI) are taken care of (Participant 6)

They also assumed that patients who are too affected by their illness do not care about being a co‐creator to prevent HAI.

The caregivers also thought that the patients were sometimes unafraid and did not think it was so important if they suffered from one more infection. Some patients were not even perceived as being oriented in the possible risks during surgery; they just want to get the surgical intervention done.

They do not think so much … these risks without …´ if only I get operated, it will be fine (Participant 1)

#### The patient has possibilities and limitations

Three categories characterise this theme; the first illustrates that *the patient's interest and resources are important to* be a co‐creator in preventing HAI. The second indicates that *the patient's previous knowledge is on different levels* concerning HAI. The last one reveals that there is both a *will and a resistance of the patient* to participate.

According to the healthcare staff, the patient's interest in preventing HAI can be seen in questions posed in connection with hospitalisation. It can be questions about hygiene in the operation theatre or how to take care of their surgical bandage when they get home. The informants also considered that many patients have the resources to be involved, in terms of the fact that they can carry out different HAI‐preventing actions while others, because of their state of health, cannot participate.

… patients with lung cancer … take care of hygiene. Yes, it depends entirely on the energy they have. Some take care of themselves (Participant 4)

The informants reflected that the patient's level of previous knowledge has an impact on the patient's ability to be a co‐creator. The staff mentioned that there is some awareness in the population about the prevalence of HAI, predominantly in the elderly generation because they have experience of nosocomial infection. Many patients have gained knowledge in the surgery intervention they will get or in the disease they have. However, many patients do not always seem to understand the fact that their own behaviour, such as lying in the bed too long, can entail a risk of infection. On the other hand, patient groups who have the prior knowledge like healthcare workers can understand how to prevent HAI.

It depends on which patient you meet. Some patients work in care and then they know about this… (Participant 2)

Interviewees believed that in many patients, there is a willingness to be independent about themselves and to follow the advice and practices that are being given in the field of hygiene measures in connection with hospital examination or treatment. Even unpleasant contraceptive measures, such as washing with bactericidal skin disinfection which can be painful, can be accepted if it is preceded by information. Patients usually also meet the healthcare personnel's decision if they are told that in some contexts there is a risk of developing an infection.

Yes, but it's not good to have. Risk of infection. … we sometimes say that to them. Then they say, "Yes, yes, let's take it out then (urinary tract catheter). (Participant 3)

Care staff also testified that there may be resistance in patients and that it may be difficult to motivate them to participate in the HAI prevention work. Some patients express that they do not want to agree on certain measures or choose to refrain, even though they have knowledge of its importance in preventing spread of infection and HAI.

… even if everyone knows that you have to wash your hands while you're on the toilet … then patients do not do that anyway (Participant 1)

There are also patients who have a different opinion than the healthcare professional in terms of HAI prevention measures and do not always follow the recommendations they received at hospital discharge; they act on their own.

## Discussion

This study describes how healthcare staff look at the patient's perquisites to be a co‐creator in preventing HAI in hospital care. The study indicates, similarly to Vaismoradi et al. 7, that there is a complex interaction between the organisation, the healthcare staff and the patient. It shows that the organisation fails to give support to enable patient participation. Furthermore, the healthcare staff find patient involvement as something new in the prevention of HAI; simultaneously, they express that they also take major responsibility for both the HAI prevention work and the patient's participation in it. At the same time, the informants state that the patient is not considered ready to be a co‐creator and is therefore given limited opportunity to participate.

### Organisation

The healthcare personnel's description, that patient participation in these contexts is something new, indicates that the hospital organisation has shortcomings in the implementation of the Patient Act 8 and the Patient Safety Act 1. The staff also highlight a lack of clear routines and practise to enable patient participation; consequently, the healthcare staff's workload, skills and perceived time pressure will control whether or not the patient is given HAI preventive information. This can be an obstacle to making the patient involved 11. Unclear organisation and role distribution create patient insecurity and barriers to the ability to be active in patient safety work 12. It can thus be concluded that, although according to both Acts 1, 8, it is the responsibility of the organisation to enable patients to participate in patient safety work. Furthermore, the result shows that there is no new way of working that enables health professionals to implement increased patient involvement. This is despite the fact that the healthcare provider has to systematically implement measures that prevent the patient from suffering during health care in accordance with the Patient Safety Act 1 and Patient Act 8. The work of making the patient a co‐creator can be perceived as a slow process, where rooted patterns dominate where the patient is seen as a recipient of care and not as a natural partner 13.

### The healthcare staff

The traditional patient safety culture mentioned above is also seen in the narratives conveyed by health professionals in this study. In addition, there is a lack of confidence in the patient's ability to be co‐creator. It has been shown that this lack of trust risks creating poorer conditions for good dialogues between staff and patient 11. Furthermore, it is argued that the patient is not considered to possess sufficient knowledge to fully assume responsibility for his/her participation 14. In accordance with Hor et al. 15, healthcare staff express therefore worries about increased risks concerning medical errors. The current study shows, however, even if healthcare professionals lack trust, they try to make the patient involved in some contexts; they consider their involvement necessary. It could be that the patient should shower at home, stop smoking before an operation or perform physical activity to prevent, for example, pneumonia in hospital care. Care staff use different communicative methods to guide the patient to this involvement. They inform and explain in a customised way where medical terms are avoided, but they also assume that they sometimes consider it necessary to both exhort and force and occasionally scare the patient into participation in preventing HAI. This may mean that they threaten the patient with the risk of infection in connection with continued inactivity and too much time in bed. However, all these methods to enhance participation in the HAI prevention work are based on one‐way communication from the healthcare staff to the patient. This is a further sign that neither the Patient Act 8 nor the Patient Safety Act 1 is fully implemented. In order to make the patient a co‐creator, dialogue is required where the patient is getting or given a place in different ways. This means that the patient must be seen as a person and respected for his or her own health and illness knowledge. Furthermore, the patient must be listened to and given the opportunity to share their views. Various communication tools can be used in support of the dialogue such as writing a diary or actively participating in documentation in the medical records 16.

### The patient

In this study, healthcare professionals expressed their perception that some patients are passive in the HAI prevention work and completely leave the responsibility to healthcare professionals. They mean that the patient does not question or take any initiative, and sometimes they seem to ignore the risk of HAI in care and treatment. However, there is reason to consider underlying causes why patients may refrain from participating in patient safety work. One might be the staffs' inhibitory attitude towards the patient expressing his/her views 13. Another could be patient age, gender or level of education where older patients, men, or patients with lower‐level education ask fewer questions 17.

The patient's health can be another obstacle to active participation; this is something which the informants in the current study address. They also highlight that there is a significant lack of knowledge among care recipients about the risks of HAI in connection with care and treatment, and they considered that this reduced the ability for the patient to be active in preventive work. It is important to continuously providing the patient with knowledge about both risks, treatment options and medicines in order to develop and participate actively and in a relevant way 13.

### Methodological considerations

Data collection for this study was conducted with staff of different sexes and ages, and from different occupational groups and clinics. This was done to get as wide representation as possible. The study was conducted only at one hospital which may have implied a limitation on representation.

However, the result is not intended for generalisation but should be seen in its context. A clear description of the study's participants, context, analysis and quotes enable the reader to determine the transferability of the results 10.

All interviews were conducted by this study's first author (EBK), who was employed at the hospital's infection control unit at the time of the survey; the function was known to the informants, which could have affected the responses. However, the importance of establishing a trustworthy relationship between the interviewer and the respondent for increased quality in the conversation is meaningful 18. In order to allow increased reliability of the data, both authors have read the transcribed text, encoded the text and then analysed together. The authors have continuously discussed the results and revised themes and categories until agreement was reached.

In order for external validation, two researchers with knowledge of the qualitative research method and the current healthcare context have also assessed the outcome of its validity 10.

## Conclusion

The patient has an important role in successful HAI prevention work and must be actively involved. Nevertheless, on an organizational level, the Patient Safety Act does not seem to be fully implemented and healthcare staff still find patient participating as something new. However, information given to the patient is considered important but still tends to happen through one‐way communication. In order to give the patient the opportunity to become co‐creator, he or she must be seen as obvious partners in HAI prevention work. An anchored, structured and systematic work centred on the patient's needs together with more support and better conditions for both healthcare professionals and patients is needed. We recommend that future studies should focus on what changes patients see as necessary to become co‐creators in the prevention of HAI in care, examination and treatment.

## Author contribution

EBK collected the data. EBK and UK planned and performed the analysis and drafted the manuscript. Both checked the manuscript for accuracy and completeness.

## Ethical consideration

The study was approved by Mid Sweden University. In accordance with Swedish legislation concerning the Ethical Review of Research Involving Humans 19, 20, this study needed no further approval. The study was conducted in compliance with the ethical principles derived from the Declaration of Helsinki 21.

The clinic administrators gave permission for the study and participants agreed to participate. The informants were given both verbal and written information about this study. Participation was voluntary, and participants could refrain at any time.

## Funding

The authors received no external financial support for the research, authorship or publication of this article.
